# Spectral peak picking improves tactile speech perception

**DOI:** 10.1038/s41598-025-28930-6

**Published:** 2025-11-24

**Authors:** Mark D. Fletcher, Carl A. Verschuur, Esma Akis

**Affiliations:** 1 Faculty of Medicine, University of Southampton, University Road, Southampton, SO17 1BJ UK; 2 University of Southampton Auditory Implant Service, University Road, Southampton, SO17 1BJ UK; 3 Institute of Sound and Vibration Research, University of Southampton, University Road, Southampton, SO17 1BJ UK

**Keywords:** Audio-tactile, Speech reading, Tactile aid, Hearing aid, Cochlear implant, Multisensory, Translational research, Perception, Auditory system, Sensory processing, Somatosensory system

## Abstract

Individual differences in speech perception often arise from disparities in access to acoustic–phonetic cues, particularly among those with hearing loss. Haptic hearing aids, which convey speech information through the sense of touch, offer a complementary pathway to improve speech understanding. However, effectively transmitting critical speech features through vibrotactile stimulation remains challenging.

To address this challenge, we introduce a tactile spectral peak picking (tSPP) approach, integrated into a vocoder-based audio-to-tactile conversion algorithm to enhance vibrotactile phoneme discrimination. The tactile vocoder decomposes audio into eight frequency bands, with tSPP selectively transmitting only the most energetic bands to emphasise dominant spectral features.

Tactile phoneme discrimination on the wrist was tested in 26 participants using either the tactile vocoder alone or with the tSPP algorithm selecting one, two, or four peaks. Discrimination improved significantly when one, two, or four peaks were selected relative to the vocoder alone, with the greatest benefits observed for one- and two-peak tSPP (average improvement: 7.5%).

These findings demonstrate that selective enhancement of spectrally salient features can improve tactile speech perception. The algorithm is suitable for real-time use in wearable sensory substitution devices and could aid the development of effective haptic hearing aids.

## Introduction

Several studies have shown that sound information can be effectively delivered through vibration to support perception of speech in quiet^1–3^ and in background noise^4–9^, as well as sound identification^10^ and localisation^11–14^ (for a review, see ref.^15^). This has potential practical application in supporting people with a hearing loss. For people using a cochlear implant, which electrically stimulates their cochlea, haptic stimulation on the wrists (“electro-haptic stimulation”^4^) has been shown to improve speech recognition in noise^4,5^ and sound localisation^11^. Neural correlates of haptic augmentation of hearing have also been identified^16–20^, contributing to a growing understanding of its neurophysiological basis. However, despite promising laboratory findings, a haptic hearing aid with proven efficacy in real-world settings has not yet been developed.

Traditionally, limitations in haptic actuator technology have severely constrained the strength and frequency range of vibrations that compact wearable devices can produce. Consequently, auditory-to-tactile sensory substitution devices have often mapped sound frequency to the spatial location of vibration on the skin (e.g.,^10,21,22^). However, these systems have either been impractically bulky^21,22^ or limited in their capacity to transfer detailed sound frequency information^10^. Recent advances in actuator design have led to the development of low-cost, compact devices capable of generating high-intensity vibration across a relatively broad frequency range with low power consumption. These advances enable a frequency-to-frequency mapping strategy to be deployed, in which sound frequency is translated to vibrotactile frequency^1,2,23,24^. This approach requires only a single actuator, allowing for a more compact device footprint while still effectively conveying frequency information, and is likely to be more intuitive than frequency-to-location mapping as it aligns more naturally with auditory perception.

A commonly used technique for converting sound into vibration is the tactile vocoder (e.g.,^4,6,10,23,25^). This approach decomposes incoming audio into frequency bands, extracts the amplitude envelope from each band, and uses these envelopes to modulate vibration intensity. In the frequency-to-frequency version of the tactile vocoder^6,23^, the amplitude envelope from each frequency band controls a different vibrotactile tone, with envelopes from lower-frequency audio bands modulating lower-frequency tones and envelopes from higher-frequency bands modulating higher-frequency tones. This approach remaps the broad audio frequency spectrum to a narrower tactile frequency range where sensitivity is highest and performance of the latest compact actuators is best. The tactile vocoder has been shown to be highly effective for transferring speech and sound location information^1,2,4–6,11,12,23,24^. Furthermore, it can readily be adapted to emphasize acoustic speech features most relevant to different user needs. For example, the latest “formant focused” version, which distributes audio bands more densely around the first and second formant frequencies, has been shown to improve transfer for vowel information^23^.

A widely used real-time method for improving spectral information transfer through a resource- or bandwidth-limited pathway is spectral peak-picking (SPP)^26–29^. SPP transmits information from only the highest-energy frequency bands in each time frame (this technique is sometimes called “number of maxima” or “*n*-of-*m*”). This prioritisation of dominant spectral components enhances intelligibility by emphasising the most perceptually salient features. When implemented in cochlear implant signal processing – a close analogue of the tactile vocoder – SPP has been shown to enhance recognition of vowels and consonants, as well as words and sentences^26,29,30^.

In the present study, we adapted the SPP method for use in a wearable haptic hearing aid. Our tactile version of SPP (tSPP) was added to the formant-focused tactile vocoder^23^ strategy, and operated with a short analysis window to enable rapid adaptation to dynamic speech cues. We assessed performance with 26 participants under four conditions: the tactile vocoder without tSPP (baseline), or the tactile vocoder with tSPP selecting either the one, two, or four most intense frequency bands within each frame. As in previous studies^1,2,23^, we evaluated tactile-only phoneme discrimination using both a male and female talker and delivered vibration to a single site at the dorsal wrist. The wrist is widely regarded as a practical and effective site for wearable haptic devices. While the fingertips provide higher vibrotactile sensitivity, the wrist offers easier self-fitting, discreet placement, a relatively large design space, and minimal interference with everyday manual activities. Moreover, previous studies have shown that the wrist has high vibration sensitivity relative to other feasible body sites, such as the arm or torso^31,32^.

We hypothesised that tSPP would enhance overall phoneme discrimination by prioritising important spectral features. Improvements were expected to be greatest for vowels because of enhanced transfer of formant structure information, which is critical to vowel perception. We also anticipated reduced performance for obstruent phonemes with one-peak tSPP, as the single frequency representation within each frame would be insufficient to convey spectral tilt and other cues critical for their discrimination.

## Results

**Fig. 1:**
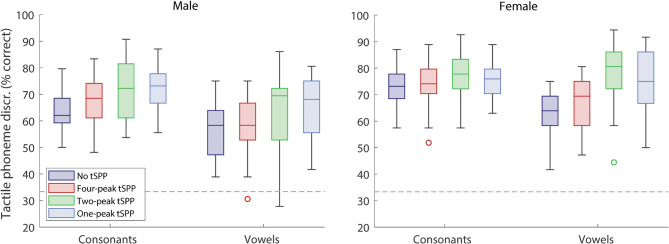
Phoneme discrimination performance for different numbers of peaks picked with tSPP. Results are shown separately for consonants and vowels and for the male (left panel) and female (right panel) talkers. Each box plot displays the median (horizontal line), upper and lower quartiles (box edges), and outliers (unfilled circles; defined as values more than 1.5 times the interquartile range). Whiskers indicate the full non-outlier range. Chance-level performance is marked by a dashed grey line.

Figure 1 shows tactile phoneme discrimination performance across four conditions: no tSPP (baseline), and tSPP with one, two, or four peaks. Across all tSPP conditions, mean phoneme discrimination performance was significantly above chance (33.3%; all *t*(25) > 21.4, all *p* < 0.001). These tests are reported for descriptive purposes only and were not corrected for multiple comparisons. A three-way repeated-measures analysis of variance (RM-ANOVA) was conducted with within-subject factors: number of peaks (eight [no tSPP], four, two, or one), phoneme type (vowel or consonant), and talker (male or female). Significant main effects were observed for the number of peaks (*F*(3,75) = 31.4, *p* < 0.001, partial η^2^ = 0.557), phoneme type (*F*(1,25) = 187.5, *p* < 0.001, η^2^ = 0.882), and talker (*F*(1,25) = 55.2, *p* < 0.001, η^2^ = 0.688). Mean phoneme discrimination accuracy was 65.1% with no tSPP (range: 51.7 to 77.8%; standard deviation (SD) = 6.7%), 67.4% with four-peak tSPP (52.8 to 78.9%; SD = 7.8%), 72.7% with two-peak tSPP (51.1 to 86.7%; SD = 9.4%), and 72.5% with one-peak tSPP (53.9 to 82.8%; SD = 7.2%). Mean discrimination was 71.8% for consonants (60.4 to 82.6%; SD = 6.5%) and 65.8% for vowels (46.2 to 77.8%; SD = 8.8%). It was 66.0% for the male talker (49.2 to 78.6%; SD = 7.9%) and 72.9% for the female talker (59.7 to 82.8%; SD = 6.8%).

Significant two-way interactions were observed between talker and both phoneme type (*F*(1,25) = 9.4, *p* = 0.005, η^2^ = 0.273) and number of peaks picked (*F*(3,75) = 3.0, *p* = 0.036, η^2^ = 0.107). No interaction was found between phoneme type and number of peaks picked. However, a significant three-way interaction was observed (*F*(3,75) = 3.2, *p* = 0.027, η^2^ = 0.115). For consonants with the male talker, mean phoneme discrimination was 61.0% with no tSPP (SD = 8.6%), 67.6% with four-peak tSPP (SD = 9.4%), 71.8% with two-peak tSPP (SD = 10.3%), and 73.0% with one-peak tSPP (SD = 7.6%). For vowels with the male talker, discrimination was 57.5% with no tSPP (SD = 10.6%), 57.8% with four-peak tSPP (SD = 11.2%), 64.0% with two-peak tSPP (SD = 13.8%), and 65.6% with one-peak tSPP (SD = 10.6%). For consonants with the female talker, discrimination was 72.5% with no tSPP (SD = 7.2%), 73.6% with four-peak tSPP (SD = 8.9%), 76.7% with two-peak tSPP (SD = 9.0%), and 75.3% with one-peak tSPP (SD = 7.0%). For vowels with the female talker, discrimination was 63.0% with no tSPP (SD = 8.6%), 67.3% with four-peak tSPP (SD = 9.5%), 76.7% with two-peak tSPP (SD = 12.3%), and 74.5% with one-peak tSPP (SD = 12.3%).

Within-subject contrasts confirmed significant improvements over baseline (no tSPP) for tSPP either with four peaks (*F*(1,25) = 6.1, *p* = 0.020, η^2^ = 0.197), two peaks (*F*(1,25) = 74.2, *p* < 0.001, η^2^ = 0.748) or one peak (*F*(1,25) = 52.2, *p* < 0.001, η^2^ = 0.676). Mean discrimination increased compared to baseline by 2.3% with four peaks (−8.3 to 9.4%; SD = 4.7%), 7.6% with two peaks (-3.3 to 14.4%; SD = 4.4%), and 7.4% with one peak (-5.6 to 13.9%; SD = 5.3%). Planned pairwise *t*-tests (with multiple comparison correction; see “Methods”) revealed greater improvement from baseline with either two- (*t*(25) = 5.9, *p* < 0.001; Cohen’s *d* = 1.1) or one-peak tSPP (*t*(25) = 5.8, *p* < 0.001; *d* = 1.0) than with four-peak tSPP. No difference was observed between one- and two-peak conditions. Contrasts showed no evidence that the change in performance from baseline for different numbers of peaks was dependent on phoneme type. Weak interactions were found between talker and the effect of tSPP compared to baseline with two peaks (*F*(1,25) = 4.5, *p* = 0.044, η^2^ = 0.152) and one peak (*F*(1,25) = 4.4, *p* = 0.047, η^2^ = 0.149), but not with four peaks.

**Fig. 2:**
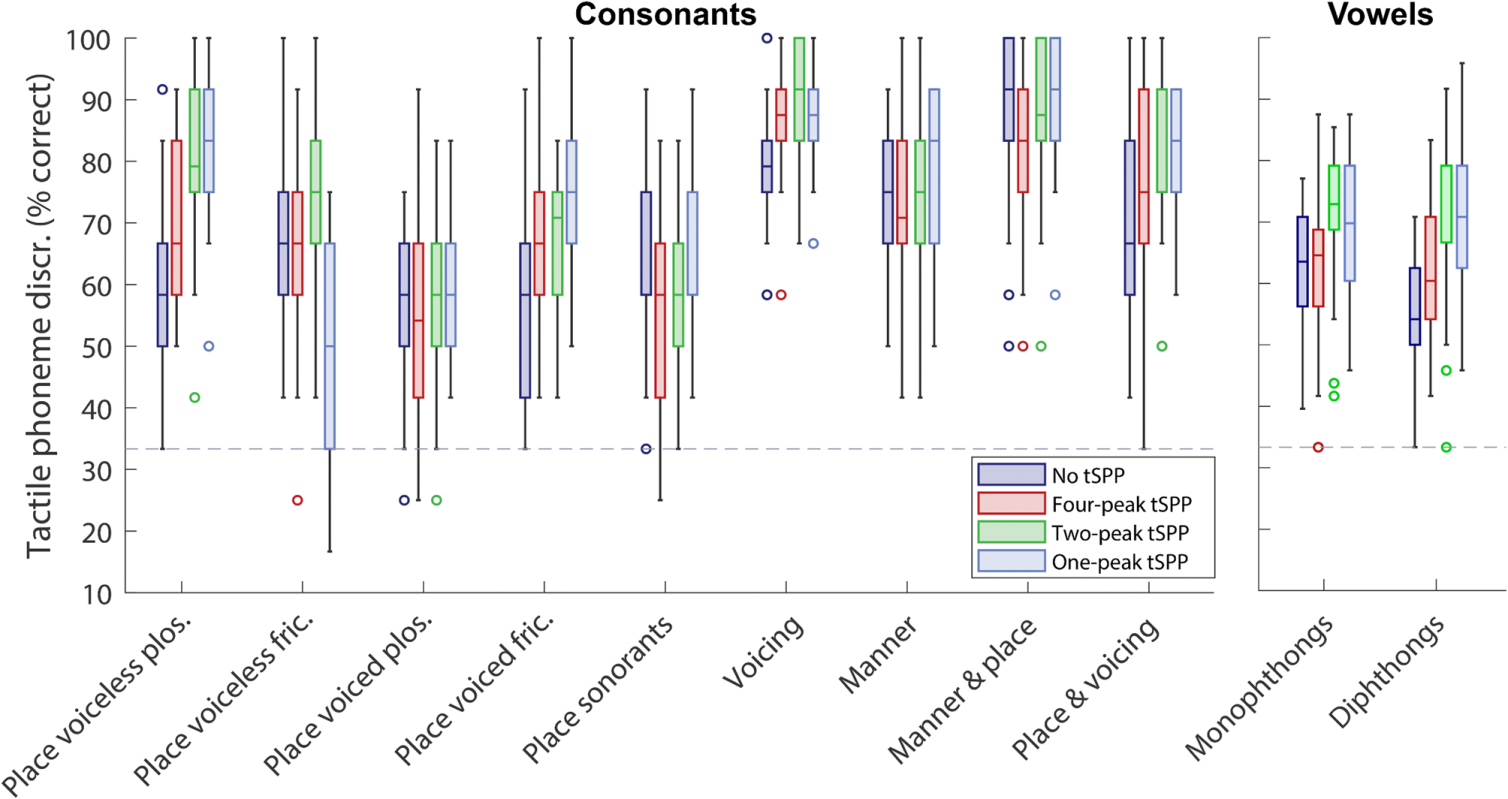
Performance for different consonant (left panel) and vowels (right panel) subgroups, under each tSPP condition. The box plots follow the format of Fig. 1.

Following the primary analyses, additional exploratory *t*-tests (with multiple comparison correction) were run to assess performance across different phoneme subgroups (see “Methods”). Figure 2 shows performance for each condition. With four-peak tSPP, a significant mean improvement of 7.2% compared to the no tSPP baseline was found for diphthong vowels (SD = 10.6%; *t*(25) = 3.5, *p* = 0.047, *d* = 0.73). With two-peak tSPP, there was a mean improvement from baseline of 17.6% for voiceless plosives differing by place of articulation (SD = 15.5%; *t*(25) = 5.8, *p* < 0.001, *d* = 1.2), of 10.6% for consonants differing by whether they were voiced (SD = 12.8%; *t*(25) = 4.2, *p* = 0.008, *d* = 1.0), and of 16.0% for consonants differing both by place of articulation and whether they were voiced (SD = 15.2%; *t*(25) = 5.4, *p* < 0.001, *d* = 1.0). There was an improvement of 6.7% for monophthong (SD = 9.7%; *t*(25) = 3.6, *p* = 0.038, *d* = 0.6) and 16.8% for diphthong (SD = 12.3%; *t*(25) = 7.0, *p* < 0.001, *d* = 1.3) vowels. For one-peak tSPP, there was a mean improvement from baseline of 17.9% for voiceless plosives differing by place of articulation (SD = 15.4%; *t*(25) = 5.9, *p* < 0.001, *d* = 1.3), 17.9% for voiced fricatives differing by place of articulation (SD = 19.0%; *t*(25) = 4.8, *p* = 0.002; *d* = 1.2), 12.8% for consonants differing both by place of articulation and whether they were voiced (SD = 17.8%; *t*(25) = 3.7, *p* = 0.030, *d* = 0.9), and 16.0% for diphthongs (SD = 12.3%; *t*(25) = 6.7, *p* < 0.001, *d* = 1.4). For voiceless fricatives differing by place of articulation, there was a decline in performance compared to baseline with one-peak tSPP of 18.9% (SD = 22.2%; *t*(25) = −4.3, *p* = 0.006, *d* = −1.2). Performance improvements were observed for both duration-matched (e.g., plosives differing by place of articulation) and non–duration-matched (e.g., diphthong) phoneme categories.

Additional post-hoc analyses (uncorrected) were conducted to explore potential predictors of phoneme discrimination (see “Methods”). The mean discrimination score across all conditions was used as the dependent variable. No evidence was found of a dependence on vibrotactile detection threshold for a tone at 125 Hz (measured during screening), participant age (spanning 20 years), or wrist circumference.

Finally, to assess whether learning or fatigue effects might have influenced performance, accuracy was compared across the repeated measurements of all phoneme pairs and tSPP conditions. These measurements were completed in sequence (with all pairs and conditions measured once before any repetitions). Mean performance across all pairs and conditions was 69.7% (SD = 6.6%) for the first repetition and 69.2% (SD = 8.2%) for the second, showing no evidence of systematic learning or fatigue effects.

## Discussion

The present study replicates previous findings that tactile phoneme discrimination on the wrist can be performed at a level significantly above chance using a formant-focused tactile vocoder (65% in the current study vs. 62% in previous work^23^, with a chance level of 33%). We further demonstrate that discrimination is significantly improved by the addition to the vocoder of one-, two-, or four-peak tSPP, with the largest improvements observed with one- and two-peak tSPP (average improvement of 7.5%). These results are particularly encouraging, as the tSPP method can be deployed on compact wearable haptic hearing aids and may prolong battery life by reducing vibration at frequencies where the actuator is less power-efficient (i.e., away from its peak resonance).

Previous work has shown that increasing the number of tactile vocoder frequency bands from one to eight improves tactile phoneme discrimination^2^, demonstrating that multichannel spectral information can be effectively transferred. The current study shows that deploying tSPP to select only a subset of the most salient spectral peaks further enhances performance. This sparser frequency representation likely reduces interference between concurrently modulating tactile tones. Because the tactile system integrates information over a relatively long temporal window, near-synchronous modulations may interact and partially mask one another, reducing the salience of key temporal features^33–35^. In addition, psychophysical work has shown that discrimination is more difficult for multiple simultaneous vibrotactile patterns than for sequential presentations or presentations with fewer patterns^36^. By selecting only the strongest peaks, tSPP may reduce this concurrent-modulation competition and allow the most informative temporal envelopes to dominate perception. Because peak selection is updated rapidly over time, temporal changes in key spectral content, such as formant transitions, can still be effectively conveyed, and may even become more salient with lower peak counts. This might explain why one- and two-peak tSPP yielded the largest improvements.

Improved transfer of formant frequency information with tSPP was expected to yield greater benefits for vowels than consonants, but no clear difference was found. However, the use of one- or two-peak tSPP substantially improved diphthong discrimination. This improvement likely reflects more accurate representation of rapid formant transitions, facilitated by the short tSPP frame lengths used.

**Fig. 3:**
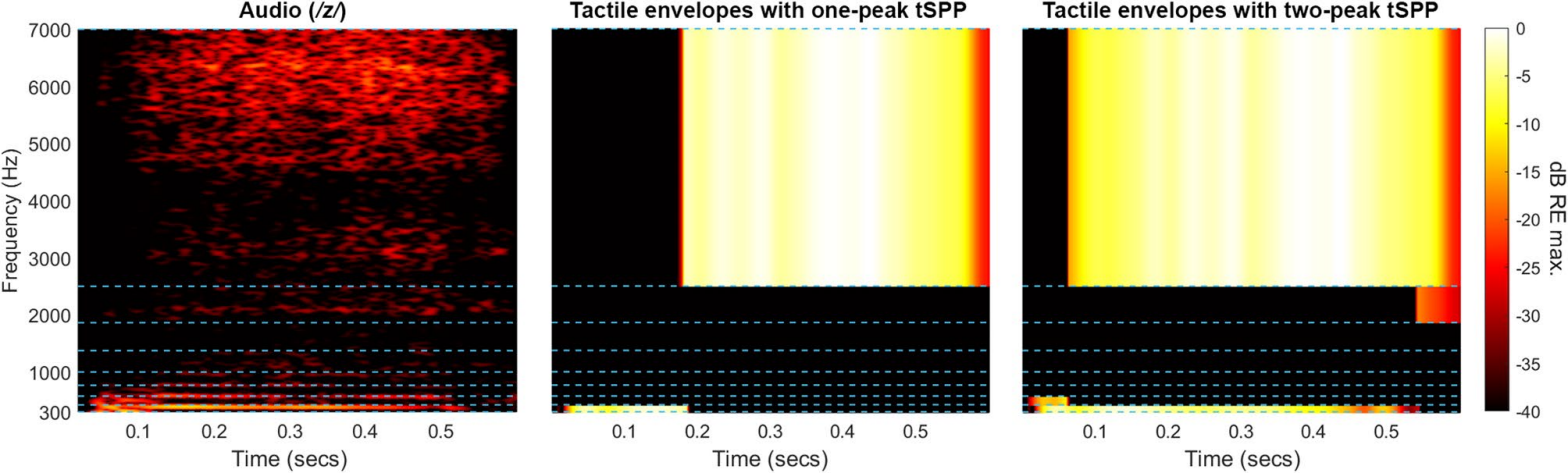
Spectrograms showing the audio (left panel) and the tactile envelopes with either one- or two-peak tSPP (central and right panels, respectively) for the voiced fricative consonant /z/, spoken by the female talker. The frequency band limits used for the tactile vocoder in the current experiment are marked with light blue dashed lines. The audio spectrogram sample rate was 22.05 kHz, and a 30-ms Hamming window with a hop size of 1 sample was used. The sample rate for the tactile spectrograms was 500 Hz, with no windowing applied. For the input audio, intensity is shown in decibels relative to the maximum magnitude of the short-time Fourier transform. For the tactile envelopes, intensity is shown in decibels relative to the maximum envelope amplitude.

Two-peak tSPP extracted more spectral information that is important to phoneme perception than one-peak tSPP. For example, discrimination of voiceless fricatives was markedly poorer with one-peak tSPP, likely due to its inability to convey spectral tilt, which is crucial for this phoneme class. Voiced fricatives illustrate this further. As shown in Fig. 3, two-peak tSPP can concurrently capture both low-frequency voicing (the lowest frequency band) and high-frequency frication noise (the highest frequency band), whereas one-peak tSPP can only capture one component within each frame. This richer spectral representation is expected to mean that two-peak tSPP offers broader utility in speech recognition tasks.

Phoneme discrimination was best for the female talker, and there was some evidence that tSPP provided greater benefit for female speech. These findings are consistent with previous research using the formant-focused tactile vocoder^23^. Improved performance with the female voice likely stems from the wider formant frequency spacing, which allows them to be better separated by the vocoder’s audio filterbank (see^23^). The addition of tSPP likely enhanced this effect by further accentuating formant peaks.

One limitation of the current study is that participants had no known hearing loss and were younger than some target users for haptic hearing aids. As tactile sensitivity tends to decline with age^37^, the generalisability of our findings could be affected. However, past studies suggest that tactile intensity discrimination and temporal gap detection for tones is robust to aging^38–40^. Moreover, some evidence suggests enhanced tactile sensitivity in individuals with congenital deafness, potentially offsetting age-related declines^41^. Importantly, prior research has found no differences in tactile speech performance between participants with and without hearing loss^2,4–6,42,43^, nor significant relationships between tactile speech performance and tactile sensitivity or age^1,2,23^. The results from the current study are therefore expected to generalise well to a range of potential haptic hearing aid users.

A second limitation is the use of phonemes rather than words or sentences. This approach was chosen to avoid the extensive training required for effective tactile word or sentence identification, which can take several months^3^. Evidence from auditory vocoder studies suggests that eight frequency bands (the number used in the current study) can transmit sufficient spectral information for recognition of “difficult” sentences^44^, and that excellent recognition is possible for “easy” sentences even with only four bands^45,46^. These findings indicate that the tactile vocoder could, in principle, support sentence-level recognition, although it remains unclear how effectively the tactile system can extract and utilise this information. While our phoneme-level task did not assess temporal boundary detection in continuous speech, previous work indicates that the tactile vocoder can convey such cues effectively^24^. Moreover, the addition of tSPP is expected to enhance the perceptual salience of phoneme boundaries by accentuating spectral transitions. This is supported by the finding of improved discrimination for both consonants and vowels, including large improvements for diphthongs, which contain pronounced spectral transitions. Future studies should examine whether these phoneme-level gains generalise to higher-level speech tasks, and whether targeted phoneme training can drive improvements in higher level speech perception.

Some earlier tactile aids, including the Tickle Talker^47^ and others^48^, extracted low-level speech features, such as fundamental frequency, using analogue zero-crossing–based methods. While attractive for real-time applications because of their computational simplicity, these approaches assume a well-defined periodic component and therefore fail for unvoiced speech and perform poorly for voiced sounds with strong noise components (e.g., fricatives or breathy vowels). They are also highly susceptible to background noise, where spurious zero-crossings can lead to erroneous estimates^49^. Our tactile-vocoder-based approach represents the distribution of acoustic energy, emphasizing spectral regions corresponding to formant energy rather than explicitly deriving features. This representation remains meaningful even when the input signal is degraded or noisy. For example, rapid changes in spectral peak selection for a noise signal still reflect the underlying acoustic reality. In contrast, traditional feature-extraction methods can produce highly misleading cues when they fail, such as indicating a high-pitched sound in response to a low-frequency noise. Modern real-time feature-extraction methods, such as PYIN^50^ or CREPE^51^, reduce estimation errors, but introduce notable algorithmic delays due to their relatively long analysis windows and computational complexity. Maintaining strong correspondence between the tactile and acoustic signal, either by directly representing acoustic energy or by minimizing feature estimation errors, is likely crucial for effective multisensory integration with vision (e.g., lip reading) and any residual hearing^52–55^. Such integration is expected to be central to the real-world effectiveness of haptic hearing aids. Future work should compare direct acoustic representation approaches with modern feature extraction methods under real-world conditions.

The tSPP algorithm selects the most prominent spectral peaks based purely on signal amplitude within each analysis frame. In clean speech, these peaks typically correspond to vocal tract resonances that signal phoneme identity across a wide range of phoneme types. In more complex or noisy acoustic scenes, peak selection likewise follows whichever frequency bands carry the strongest energy. When interfering sounds are weaker than the target speech (as in most real-world listening scenarios^56^), tSPP suppresses them; however, noise components will be selected if they dominate the spectrum. Robustness to background noise or competing sounds may be improved through more advanced band-selection strategies (e.g., ^57,58^) or by incorporating traditional^59^ or state-of-the-art noise-reduction methods^24^ (perhaps in combination with advanced band-selection approaches).

Tailoring the tactile vocoder to individual users may also enhance performance. For cochlear implant users, who often lack access to sound below ~ 300 Hz^60^, frequency bands could be shifted towards low frequencies (as in^4^). In this case, one-peak tSPP might be considered to most effectively transfer voicing and fundamental frequency cues, or feature extraction methods might be deployed (e.g.,^21,61^). Conversely, users with functional low-frequency hearing but a substantial high-frequency loss might benefit from frequency bands being shifted towards high-frequencies. For those who receive little or no useful information through audition, such as individuals with profound hearing loss who cannot use cochlear implants, optimising the vocoder for lip-reading support may be beneficial. This could involve selecting frequency bands that provide complementary low-frequency voicing and high-frequency fricative/plosive cues^62^. In such cases, multiple-peak tSPP would be necessary to transmit this information concurrently (as in the example of the voiced fricative shown in Fig. 3).

Beyond frequency-band targeting, individualisation could include tailoring the tactile stimulation profile by adjusting gain or compression applied to tactile tones based on a user’s sensitivity or preferences. This could be achieved through clinical protocols adapted from hearing aid or cochlear implant fitting strategies (e.g., DSL^63^ or NAL^64^), or advanced over-the-counter self-fitting tools like EarTuner or Camadapt^65^. However, even coarser self-fitting approaches, such as the “Goldilocks” method in which the user can adjust only the broadband or low and high frequency gain settings^66,67^, have been shown to be highly effective^68^.

In conclusion, this study provides strong evidence that tSPP significantly enhances tactile phoneme discrimination with the tactile vocoder strategy. The method can be readily deployed in real-time on a compact device and therefore holds substantial promise for future haptic hearing aids. The findings of the current study are particularly exciting, as tSPP is likely to have utility for a range of tactile vocoder designs that focus on different speech features to maximise benefit for different clinical groups. Just as SPP has proven critical for optimizing cochlear implant performance, tSPP may play a central role in maximising speech understanding through haptic hearing aids.

## Methods

### Participants

Table 1 presents characteristics for the 26 participants (13 male, 13 female) who took part in this study. Participants were aged 18–37 years (with a mean age of 26 years). All reported no known issues with touch perception, as confirmed via a health questionnaire, and demonstrated normal vibrotactile sensitivity, with detection thresholds at the index fingertip below 0.3 m/s^2^ RMS for a sinusoid at 31.5 Hz and below 0.7 m/s^2^ RMS for a sinusoid at 125 Hz (see “Procedure” for details).

**Table 1:** Participant characteristics. For each participant, the table shows: vibrotactile detection thresholds at the fingertip (measured during screening); wrist temperature (measured during testing); wrist height, width, and circumference; dominant hand; age; and biological sex. The mean and standard deviation (SD) across participants is also shown.

ID	31.5 Hz thresh. (m/s^-2^)	125 Hz thresh. (m/s^-2^)	Wrist temp. (°C)	Wrist height/ width (mm)	Wrist circum. (mm)	Dom. hand (L/R)	Age (yrs.)	Sex (M/F)
1	0.05	0.07	30.5	52/41	160	R	29	M
2	0.02	0.08	30.4	39/58	166	R	36	M
3	0.06	0.10	31.7	36/51	150	R	29	F
4	0.04	0.10	33.5	48/31	139	R	27	F
5	0.03	0.07	31.0	36/48	149	L	28	F
6	0.06	0.06	32.1	45/60	190	R	31	M
7	0.17	0.17	30.6	34/49	144	R	22	F
8	0.02	0.03	32.6	35/51	148	R	28	F
9	0.05	0.04	29.7	45/52	167	R	22	M
10	0.03	0.03	29.6	46/57	177	R	38	M
11	0.03	0.03	33.0	34/47	137	R	36	F
12	0.05	0.02	30.7	37/49	169	R	27	F
13	0.07	0.07	29.5	36/51	155	R	22	M
14	0.03	0.10	21.8	34/47	135	R	28	F
15	0.05	0.06	31.0	42/54	162	R	18	M
16	0.06	0.24	30.7	34/48	143	R	21	M
17	0.02	0.06	30.6	33/46	138	R	21	F
18	0.06	0.03	29.1	41/53	174	R	22	M
19	0.04	0.06	29.9	46/47	156	L	21	M
20	0.08	0.16	31.4	41/53	161	R	22	M
21	0.03	0.05	31.0	34/47	140	R	28	F
22	0.05	0.09	30.5	31/44	142	R	32	F
23	0.05	0.09	33.6	39.53	164	R	19	M
24	0.05	0.03	29.3	44/59	171	R	19	M
25	0.05	0.06	30.9	39/61	170	R	31	F
26	0.10	0.16	31.6	34/42	139	R	22	F
Mean	0.05	0.08	30.6	39/50	156	–	26	–
SD	0.03	0.05	2.2	5/6	15	–	6	–

The wrist circumferences in our participant sample were broadly representative of the general adult population, with the average slightly below that expected in the population, the smallest reaching the 2nd percentile for females, and the largest reaching the 97th percentile for males (based on U.S. military anthropometric data)^69^.

No participants reported any hearing problems. All were recruited from university staff and students and provided informed consent prior to participation. Participants received £20 for taking part in the study.

### Stimuli

The experimental procedure followed the methodology of previous studies^1,23^. The tactile stimulus used for the phoneme discrimination task was generated using the EHS Research Group Phoneme Corpus^2^. This contains recordings of a British English male and female talker producing each of the 44 UK British English phonemes, with four recordings of each phoneme per talker. A subset of 45 phoneme pairs was selected, each containing four recordings of each phoneme for both the male and female talker (360 recordings in total). These pairs matched those used in previous work^1,23^, and were chosen to maximize functional relevance for potential haptic hearing aid users while maintaining a wide range of phonemic contrasts based on differences in frequency, amplitude and temporal information (see Table 2).

**Table 2:** Consonant and vowel pairs used in the experiment, grouped by the type of contrast. Examples of the British English phonemes (bold and underlined) being used in words are also shown (note that these words are for illustration only and were not used in testing).

Consonants		Contrast type	Vowels		Contrast type
*t & p*	*(****t****ea/****p****en)*	*Place in voiceless plosives*	*ɪ & ɑː*	*(k****i****t/c****ar****t)*	*Monophthongs*
*t & k*	*(****t****ea/****k****ey)*	*Place in voiceless plosives*	*iː & æ*	*(s****ea****/b****a****d)*	*Monophthongs*
*k & p*	*(****k****ey/****p****en)*	*Place in voiceless plosives*	*ɔː & ɪ*	*(l****aw****/k****i****t)*	*Monophthongs*
*f &* θ	*(****f****at/pa****th****)*	*Place in voiceless fricatives*	*ʊ & ɑː*	*(p****u****t/c****ar****t)*	*Monophthongs*
*f & s*	*(****f****at/****s****un)*	*Place in voiceless fricatives*	*uː & ʌ*	*(bl****ue****/m****u****d)*	*Monophthongs*
*ʃ & s*	*(****sh****e/****s****un)*	*Place in voiceless fricatives*	*æ & e*	*(b****a****d/b****e****d)*	*Monophthongs*
*d & b*	*(****d****ay/****b****ay)*	*Place in voiced plosives*	*ʊ & ɪ*	*(p****u****t/k****i****t)*	*Monophthongs*
*g & d*	*(****g****et/****d****ay)*	*Place in voiced plosives*	*æ & ɒ*	*(b****a****d/l****o****t)*	*Monophthongs*
*g & b*	*(****g****et/****b****ay)*	*Place in voiced plosives*	*iː & uː*	*(s****ea****/bl****ue****)*	*Monophthongs*
*v & ð*	*(****v****et/****th****is)*	*Place in voiced fricatives*	*ʌ & æ*	*(m****u****d/b****a****d)*	*Monophthongs*
*v & z*	*(****v****et/****z****oo)*	*Place in voiced fricatives*	*uː & ʊ*	*(bl****ue****/p****u****t)*	*Monophthongs*
*ð & z*	*(****th****is/****z****oo)*	*Place in voiced fricatives*	*iː & e*	*(s****ea****/b****e****d)*	*Monophthongs*
*l & r*	*(****l****ot/****r****un)*	*Place in sonorants*	*ɔɪ & eɪ*	*(b****oy****/d****ay****)*	*Diphthongs*
*j & l*	*(****y****et/****l****ot)*	*Place in sonorants*	*ɔɪ & aʊ*	*(b****oy****/n****ow****)*	*Diphthongs*
*m & n*	*(****m****en/****n****ot)*	*Place in sonorants*	*aʊ & eɪ*	*(n****ow****/d****ay****)*	*Diphthongs*
*z & s*	*(****z****ero/****s****un)*	*Voicing*	*ɪə & əʊ*	*(n****ear****/n****o****)*	*Diphthongs*
*ʒ & ʃ*	*(vi****s****ion/****sh****e)*	*Voicing*	*ʊə & eɪ*	*(p****oor****/d****ay****)*	*Diphthongs*
θ *& ð*	*(pa****th****/****th****is)*	*Voicing*	*eə & ʊə*	*(f****air****/p****oor****)*	*Diphthongs*
*t & s*	*(****t****ea/****s****un)*	*Manner*			
*b & w*	*(****b****ay/****w****et)*	*Manner*			
*tʃ & ʃ*	*(****ch****at/****sh****e)*	*Manner*			
*ð & b*	*(****th****is/****b****ay)*	*Manner & place (two-feature)*			
*k & s*	*(****k****ey/****s****un)*	*Manner & place (two-feature)*			
*g & r*	*(****g****et/****r****un)*	*Manner & place (two-feature)*			
*v & s*	*(****v****et/****s****un)*	*Place & voicing (two-feature)*			
θ* & z*	*(pa****th****/****z****ero)*	*Place & voicing (two-feature)*			
*m & v*	*(****m****en/****v****et)*	*Place & voicing (two-feature)*			

To prevent total duration from serving as a discrimination cue, most phoneme pairs were duration-matched by applying a 20-ms raised-cosine fade-out, reaching zero amplitude at the end of the shortest stimulus (defined as the point where the signal amplitude dropped below 1% of its maximum). However, duration matching was not applied to diphthongs or to phoneme pairs containing */g/, /d/, /l/, /r/, /v/, /w/,* or */j/,* because these phonemes cannot be produced in isolation without altering their acoustic characteristics. Duration could therefore have served as a discrimination cue only for diphthongs and for phoneme pairs containing these phonemes.

**Fig. 4:**
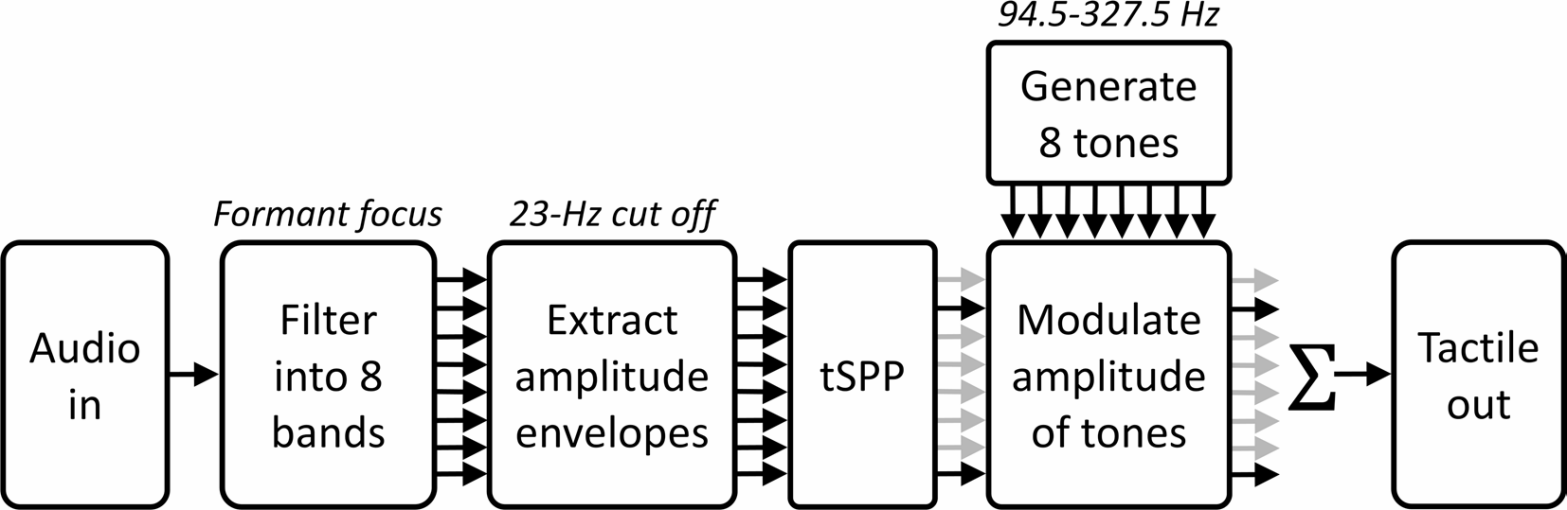
Block diagram showing the tactile vocoder signal-processing chain used to convert sound into vibration.

Audio was converted to vibrotactile stimulation using a tactile vocoder strategy similar to that used in previous studies^1,2,4–6,11,12,23,24^ (see Fig. 4). The audio intensity was normalised using ITU P.56 method B^70^ to ensure consistent signal energy across phonemes. The signal was downsampled to 16,000 Hz, matching the sampling rate of many hearing aids and other compact real-time audio devices. The signal was then passed through a 512th-order FIR filter bank with eight bands, using the previously developed “formant-focused” approach^23^. Four bands were spaced between 300 and 1,000 Hz (targeting formant 1), three were spaced between 1,000 and 2,500 Hz (targeting formant 2), and one was between 2500 and 7000 Hz (retaining high-frequency information critical for obstruent phoneme discrimination). Within these frequency ranges, all bands were equally spaced on the equivalent rectangular bandwidth (ERB) scale^71^.

After band-pass filtering, the amplitude envelope was extracted for each band using a Hilbert transform and a zero-phase 6th-order low-pass Butterworth filter, with a corner frequency of 23 Hz (following Fletcher et al.^2^). In conditions with tSPP active, only the highest-energy bands were used, with one, two, or four bands selected per trial. The band selection was updated every 6 ms. The envelopes for the active bands were applied from the first zero-crossing of the corresponding vibrotactile carrier tone, while envelopes for inactive bands were set to zero.

The amplitude envelopes were used to modulate the amplitudes of eight fixed-phase vibrotactile sinusoidal carriers with frequencies of 94.5, 116.5, 141.5, 170, 202.5, 239, 280.5, and 327.5 Hz. The frequencies were centred on 170 Hz, the frequency at which vibration output peaks for many compact haptic actuators. The spacing was determined based on frequency discrimination thresholds measured at the dorsal forearm^72^, as equivalent data for the wrist are currently unavailable. The frequency range was deliberately limited to: (i) include frequencies reproducible by compact, low-power actuators suitable for integration into future wrist-worn haptic devices intended for everyday use; (ii) fall within the region of greatest human tactile sensitivity; and (iii) remain minimally audible, thereby avoiding distraction or annoyance for nearby individuals and users with residual hearing.

To compensate for differences in vibrotactile sensitivity across frequencies, frequency-specific gain adjustments were applied to each vibrotactile carrier tone. As in previous work^1,2,23,24^, these were derived based on previous detection threshold measurements^73^. The gains were 13.8, 12.1, 9.9, 6.4, 1.6, 0, 1.7, and 4 dB, respectively. The eight carrier tones were summed and delivered through vibrotactile stimulation at a single contact point. The tactile stimuli were scaled to have an equal RMS amplitude, yielding a nominal output level of 1.2 G (141.5 dB ref. 10^–6^ m/s^2^), a level that can be produced by a range of compact, low-powered haptic actuators. The stimulus level was roved by ± 3 dB (uniform distribution) to prevent phoneme discrimination based on absolute intensity cues. To mask residual auditory cues, pink noise was presented through headphones at 60 dBA.

### Apparatus

Participants were seated throughout the experiment in a vibration-isolated, temperature-controlled room with an average temperature of 23 °C (SD = 0.45 °C). Room and participant skin temperatures were measured using a Digitron 2022 T Type K thermocouple thermometer. The thermometer was calibrated in accordance with ISO 80,601-2-56:2017^74^, following the procedure described by Fletcher et al.^2^. Control of skin temperature was necessary because vibrotactile sensitivity is known to vary with temperature^75^.

During screening, vibrotactile detection thresholds were measured using an HVLab Vibrotactile Perception Meter^76^ with a circular 6-mm diameter probe. The probe gave a constant upward force of 1N and had a rigid surround. A downward force sensor was built into the surround, and the applied force was displayed to the participant. The sensor was calibrated using Adam Equipment OIML calibration weights. Vibration intensity was calibrated using the Vibrotactile Perception Meter’s built-in accelerometers (Quartz Shear ICP, model 353B43) and a Brüel & Kjær (B&K) Type 4294 calibration exciter. All stimuli had a total harmonic distortion of < 0.1% and the system conformed to ISO-13091–1:2001^77^.

**Fig. 5:**
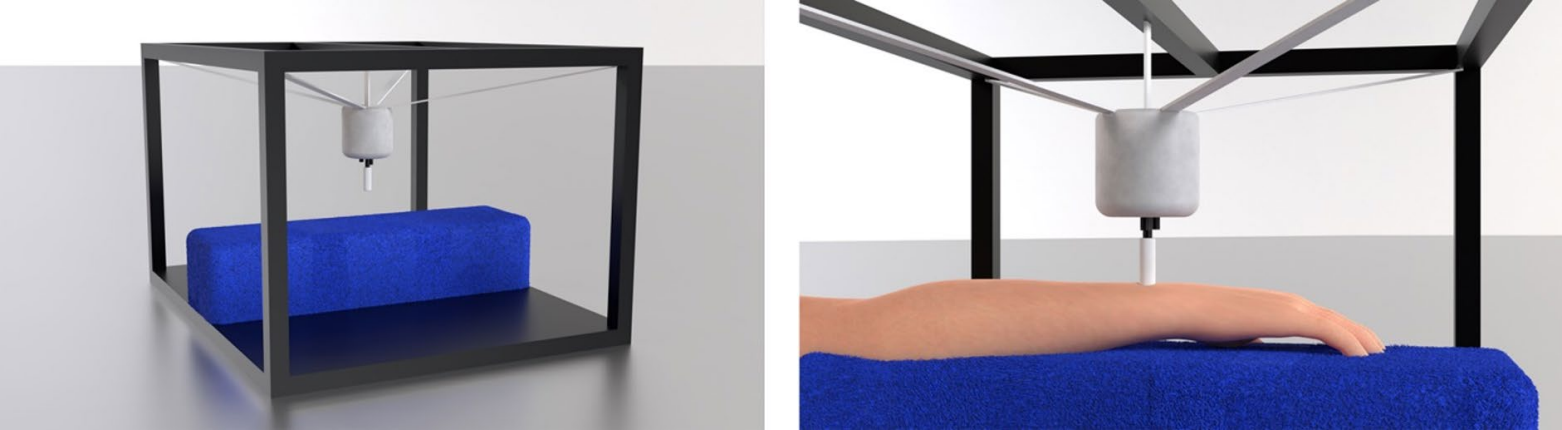
3D renders of the EHS Research Group haptic stimulation rig. Left: the rig without the participant’s arm. Right: close-up view with the participant’s arm resting on the blue foam cushion and the shaker probe contacting the dorsal wrist. Image reproduced from Fletcher et al.^2^ with permission of the authors.

In the experiment phase, vibrotactile stimulation was delivered using the EHS Research Group haptic stimulation rig (see Fig. 5)^2^. This comprised a Ling Dynamic Systems V101 shaker with a custom 10-mm diameter circular probe, 3D-printed from Verbatim polylactic acid (PLA). The shaker was driven by a MOTU UltraLite-mk5 sound card, an RME QuadMic II preamplifier, and an HVLab Tactile Vibrometer power amplifier. The shaker was suspended in an adjustable elastic cradle from an aluminium strut frame, with the probe oriented downward to contact the dorsal wrist. The participant’s palmar forearm rested on a 95-mm-thick foam cushion positioned below the probe. The probe applied a downward force of 1 N, calibrated for each participant using a B&K UA-0247 spring balance. Vibration output was calibrated using a B&K 4533-B-001 accelerometer and B&K Type 4294 calibration exciter. All experimental stimuli had a total harmonic distortion of < 0.1%.

Masking audio was played from the MOTU UltraLite-mk5 sound card through Sennheiser HDA 300 sound-isolating headphones. Audio levels were calibrated using a B&K G4 sound level meter with a B&K 4157 occluded ear coupler. The sound level meter was calibrated using a B&K Type 4231 calibrator.

### Procedure

Each participant completed the experiment in a single session lasting approximately two hours. Informed consent was obtained before testing, and participants completed a screening questionnaire to confirm that they (1) did not have any condition affecting tactile sensitivity, (2) had not experienced injury or undergone surgery on the hands or arms, and (3) had not been exposed to intense or prolonged hand or arm vibration within the previous 24 h. Skin temperature was then measured at the index fingertip of the dominant arm, and participants proceeded only when their skin temperature fell between 27 °C and 35 °C.

Vibrotactile detection thresholds were then measured at the index fingertip, following BS ISO 13,091-1:2001^77^. During the threshold measurements, participants applied a downward force of 2 N, monitored via the Vibrotactile Perception Meter display. Participants were required to demonstrate touch perception thresholds within the normal range (< 0.3 m/s^2^ RMS at 31.5 Hz and < 0.7 m/s^2^ RMS at 125 Hz)^78^. The fingertip was used for screening because normative data are not currently available for the wrist. If participants passed all screening phases, their wrist dimensions (at the position they would typically wear a wristwatch) were recorded before they progressed to the experiment phase.

In the experiment phase, participants sat in front of the EHS Research Group haptic stimulation rig (Fig. 5), with the forearm of their dominant arm supported on the foam cushion. The shaker probe was adjusted to contact the centre of the dorsal wrist (again, at the position typically used for a wristwatch). Skin temperature was required to be between 27 °C and 35 °C before testing commenced.

Participants completed a previously developed three-interval, three-alternative forced-choice phoneme discrimination task^2^. On each trial, one phoneme pair spoken by either the male or female talker was presented (see “Stimuli”). Two intervals contained one phoneme from the pair (selected at random), and one interval contained the other phoneme. Intervals were separated by 250 ms, and their order was randomized on each trial. Participants identified via keypress (with their non-dominant hand) which interval contained the oddball phoneme (i.e., the phoneme presented only once). Task instructions were given, including an instruction to disregard the overall vibration intensity, as level roving was applied to prevent use of overall intensity as a discrimination cue. Participants had the opportunity to ask questions if they did not understand. No additional training or familiarisation was completed as previous work has shown that this is not required for this task^23^. Visual feedback was displayed for 500 ms after each trial to indicate whether the response was correct or incorrect.

Phoneme discrimination accuracy was measured for four conditions: no tSPP, or one-, two-, or four-peak tSPP. For each condition, all phoneme pairs (see “Table 2”) were tested with both the male and female talker. Each phoneme pair was presented twice per talker, with the phoneme sample randomly selected from the four samples available in the corpus, meaning a total of 720 trials per participant. For each participant, all phoneme pairs and conditions were measured once in a random order before the repeat measurements were conducted, with the order of trials re-randomised for the repetition.

The study protocol was approved by the University of Southampton Faculty of Engineering and Physical Sciences Ethics Committee (ERGO ID: 68477). All procedures were conducted in accordance with relevant ethical guidelines and regulations.

### Statistics

The primary analysis employed a three-way repeated-measures analysis of variance (RM-ANOVA) with three factors: number of tSPP peaks (eight [no tSPP], four, two, or one), phoneme type (consonant or vowel), and talker (male or female). Data were determined to be normally distributed based on visual inspection and on Kolmogorov–Smirnov and Shapiro–Wilk tests. The significance level (α) was set at 0.05. Within-subject contrasts compared performance with four-, two-, and one-peak tSPP against the no-tSPP baseline. Planned two-tailed *t*-tests (Bonferroni-Holm corrected^79^ for multiple comparisons) were used to compare overall phoneme discrimination between the four-peak tSPP condition and the one- and two-peak conditions, and between two-peak and one-peak tSPP.

Exploratory *t*-tests (Bonferroni-Holm corrected for 36 comparisons) were conducted to evaluate phoneme discrimination for individual phoneme subgroups (see “Table 2”) across the one-, two-, and four-peak tSPP conditions relative to no tSPP. Finally, post hoc Pearson correlation analyses assessed relationships between phoneme discrimination accuracy and (1) vibrotactile detection thresholds at 125 Hz (measured at screening), (2) participant age, and (3) wrist circumference. No correction for multiple comparisons was applied to these exploratory correlation analyses, as no significant associations were predicted based on previous findings^1,2,23^.

A priori power analysis was conducted based on a published study (N = 20) that used the same tactile phoneme discrimination task and a similar within-subject design^23^ (albeit with a different number of conditions and experimental manipulations). The analysis considered all six planned within-subject comparisons (baseline vs. each tSPP condition, and pairwise comparisons between tSPP conditions). Paired-comparison contrasts used an SD of 3.46% and difference-of-differences contrasts used an SD of 3.18%. The power analysis calculated the required N for each contrast separately and selected the maximum requirement across contrasts, yielding a study-wide sample size of 24 participants to achieve 80% power (per-comparison α = 0.0083, Bonferroni-corrected) for detecting a 2.5-percentage-point improvement in phoneme discrimination. The final sample size of 26 exceeded this requirement, providing sufficient sensitivity to detect both moderate and large effects across all planned comparisons. Observed effects for the one- and two-peak tSPP conditions (7.4–7.6 percentage point improvements) were well above this threshold, while the four-peak condition (2.3 points) was near the detection threshold, consistent with its moderate significance.

## Data Availability

The datasets generated and analysed during the current study are available in the University of Southampton’s Research Data Management Repository at: 10.5258/SOTON/D3763.
